# The *Arabidopsis BLAP75/Rmi1* Homologue Plays Crucial Roles in Meiotic Double-Strand Break Repair

**DOI:** 10.1371/journal.pgen.1000309

**Published:** 2008-12-19

**Authors:** Liudmila Chelysheva, Daniel Vezon, Katia Belcram, Ghislaine Gendrot, Mathilde Grelon

**Affiliations:** INRA de Versailles, Institut Jean-Pierre Bourgin, Station de Génétique et d'Amélioration des Plantes UR-254, Versailles, France; National Cancer Institute, United States of America

## Abstract

In human cells and in *Saccharomyces cerevisiae*, BLAP75/Rmi1 acts together with BLM/Sgs1 and TopoIIIα/Top3 to maintain genome stability by limiting crossover (CO) formation in favour of NCO events, probably through the dissolution of double Holliday junction intermediates (dHJ). So far, very limited data is available on the involvement of these complexes in meiotic DNA repair. In this paper, we present the first meiotic study of a member of the BLAP75 family through characterisation of the *Arabidopsis thaliana* homologue. In *A*. *thaliana blap75* mutants, meiotic recombination is initiated, and recombination progresses until the formation of bivalent-like structures, even in the absence of ZMM proteins. However, chromosome fragmentation can be detected as soon as metaphase I and is drastic at anaphase I, while no second meiotic division is observed. Using genetic and imunolocalisation studies, we showed that these defects reflect a role of *A. thaliana* BLAP75 in meiotic double-strand break (DSB) repair—that it acts after the invasion step mediated by RAD51 and associated proteins and that it is necessary to repair meiotic DSBs onto sister chromatids as well as onto the homologous chromosome. In conclusion, our results show for the first time that BLAP75/Rmi1 is a key protein of the meiotic homologous recombination machinery. In *A*. *thaliana*, we found that this protein is dispensable for homologous chromosome recognition and synapsis but necessary for the repair of meiotic DSBs. Furthermore, in the absence of BLAP75, bivalent formation can happen even in the absence of ZMM proteins, showing that in *blap75* mutants, recombination intermediates exist that are stable enough to form bivalent structures, even when ZMM are absent.

## Author Summary

Recombination is a process by which cells can repair DNA damage. Such repair can either be crossovers (CO), in which DNA molecules are submitted to major exchanges, or non-crossover (NCO) events. Eukaryotic cells have developed several mechanisms to maintain genome stability during vegetative development by limiting the occurrence of CO events in favour of NCO. BLAP75/Rmi1, BLM/Sgs1, and TopoIIIα/Top3 act together in a complex (BTB/RTR) known to be a crucial component of regulation mechanisms against CO formation. However, CO/NCO regulation is thought to be very different during meiosis since homologous chromosomes (paternal and maternal) overcome at least one CO/pair. In this study, we investigate the role of the BTB/RTR complex during meiotic recombination through the analysis of a function of one of its members: the *A*. *thaliana* homologue of BLAP75/Rmi1. We show for the first time that BLAP75/Rmi1 is also a key protein of the meiotic homologous recombination machinery. In *Arabidopsis*, we found that this protein is dispensable for homologous chromosome recognition and synapsis, but necessary for the repair of meiotic double-strand breaks. Furthermore, in the absence of BLAP75, bivalent formation can happen even in the absence of CO.

## Introduction

From a diploid mother cell, meiosis generates four haploid products from which gametes differentiate. This ploidy reduction is a direct consequence of two rounds of chromosomal segregation (meiosis I and meiosis II) following a single S phase. The first meiotic division separates homologous chromosomes from each other while meiosis II separates sister chromatids.

Recombination is one of the key events in meiosis. It gives rise to crossovers (COs), which are essential for the correct segregation of homologous chromosomes during meiosis I, ensuring the association of homologous chromosomes into bivalents. Meiotic recombination can also lead to gene conversion not associated with COs (NCOs), events that are probably much more frequent than COs at least in plants and mammals [1].

The current model for meiotic recombination [2],[3] proposes that it is initiated by the programmed formation of DNA double-strand breaks (DSBs), which are then resected to generate 3′ single stranded DNA molecules that drive DNA repair onto the homologous chromosome by invading an intact homologous chromosome. DNA strand exchange results in the formation of joint molecules. These joint molecules either dissociate enabling the broken chromosome to rejoin through synthesis-dependent strand annealing (SDSA) [3]–[5], or form stable D-loops which proceed through the capture of the second processed DNA end to produce a double Holliday junction intermediate (dHJ). The dHJ is then resolved by an unknown resolvase to generate COs products. This CO pathway is under the control of a set of genes which includes the *ZMM* family (for Zip1, Zip2, Zip3, Zip4, Mer3 and Msh4/Msh5). Another CO pathway, that does not proceed through dHJ formation, also coexists in most species and is under the control of the Mus81/Mms4 endonuclease [6].

In somatic cells, homologous recombination (HR) is also used to repair DNA DSBs that arise either from damage or from stalled replication forks. In this context, contrary to what happens during meiotic HR, repair is mostly directed towards the sister chromatids rather than the homologous chromosome. Furthermore, COs are generally prevented in favour of NCO events, probably by preferential involvement of the SDSA repair pathway and to dissolve dHJ to generate NCO events.

The eukaryotic homologues of the highly conserved RecQ helicase family are known to be particularly crucial components of regulation mechanisms against CO formation. The Bloom protein (BLM, one of the five human RecQ helicases) was shown to disrupt D-loop intermediates *in vitro* [7], to dissolve dHJ [8],[9] and to disrupt the Rad51 presynaptic filament [9],[10]. *In vivo*, the antiCO effect of BLM/Sgs1 helicase was demonstrated by the fact that yeast *sgs1* mutants as well as Bloom's syndrome patients or BLM-deficient mice have elevated rates of mitotic recombination (either reciprocal sister chromatid exchanges (SCE) or increased frequency of exchange between homologous chromosomes) [11]–[14]. In plants at least seven *RECQ-like* genes were identified [15] and functional analyses showed that *A. thaliana RECQ4A* is likely to be the functional homologue of *BLM* [16],[17]. It was also shown to partially suppress the embryo-lethality of *A. thaliana top3α* and to be lethal in conjunction with the *A. thaliana mus81A* mutation [18].

The human protein BLAP75 (for Bloom Associated Protein of 75 kD) was recently identified [19],[20] as a 75 kD protein which co-purified in diverse Bloom (BLM)-containing complexes from HeLa cells. It was proposed to form the structural core of all BLM complexes with BLM and TopoIIIα (the human topoisomerase 3α) [19]. A BLAP75 homologue was described in yeast (Rmi1/Nce4) and the conservation of the BTB complex (BLM-TopoIII-BLAP75) was demonstrated in *S. cerevisiae*, where it is known as the RTR (RecQ helicase-Top3-Rmi1) complex [21],[22]. Recent biochemical studies showed that the BTB/RTR complex plays a crucial role in the dissolution of dHJ to produce NCOs [9],[23],[24]. The proposed mode of action is that BLM/Sgs1 decatenates dHJs to form a hemicatenane substrate for the topoisomerase 3. BLAP75/Rmi1 would be necessary for the loading and stability of the complex. It strongly enhances BLM-TopoIIIα dependant dHJ dissolution *in vitro* [9], [21], [24]–[26]. It is also possible that this complex works on other HR substrates such as Rad51 presynaptic filaments or D loops [10],[27]. Therefore, the BTB/RTR complexes are proposed to act at different levels of the HR process to limit CO formation in favour of NCO events.

Limited data is available on the involvement of BTB/RTR complexes in meiotic DNA repair. In *S. cerevisiae, sgs1*Δ *top3*Δ, and *rmi*Δ mutants show reduced sporulation and decreased spore viability [11],[12],[22],[28],[29]. For *sgs1*, the phenotype was correlated with meiosis I nondisjunction and precocious sister segregation [11],[28] but, unlike the situation in somatic cells, in most cases no increase in meiotic recombination was detected [11],[28],[30],[31]. Nevertheless, Sgs1 was shown to prevent CO maturation in *zmm* mutants, and could suppress sister chromatid dHJ formation during meiotic recombination [30],^31^. In mouse spermatocytes, the BLM protein was shown to colocalise with the recombination proteins RPA, RAD51/DMC1 and MSH4 [32]–[34], but *BLM* disruption in mouse has no effect on meiotic CO rates [14]. This is not the case for the *Drosophila melanogaster*, *Caenorhabditis elegans* and *Schizosaccharomyces pombe BLM* orthologues, for which depletion is associated with a decrease in CO rates [35]–[37].

In this study we show that the *Arabidopsis BLAP75/Rmi1* homologue is absolutely required for meiotic DSB repair onto homologous chromosome or sister chromatid. We also provide evidence that, in the absence of *A. thaliana BLAP75*, recombination is initiated and progresses until the formation of recombination intermediates that allow the formation of bivalents even in the absence of ZMM.

## Results

### Identification and Molecular Characterisation of *A. thaliana BLAP75/RMI1*

**Figure 1:**
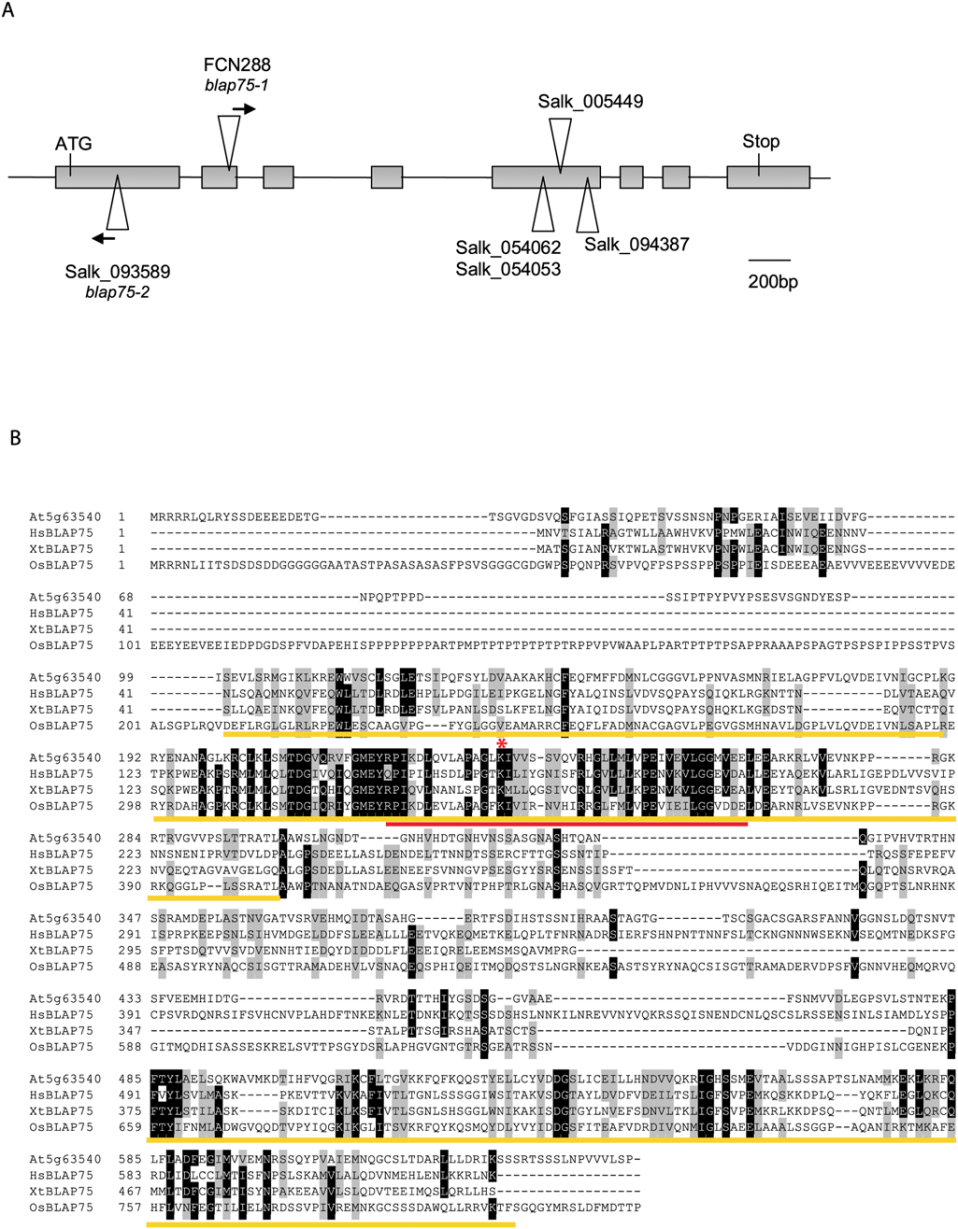
The *A. thaliana BLAP75/RMI1* open reading frame. (A) Schematic representation of the *A. thaliana BLAP75/RMI1* coding sequence. Exons are represented as grey boxes and T-DNA insertions in the studied alleles are indicated. Arrows show the orientation of the sequenced T-DNA left border. (B) Alignment of *A. thaliana* (At5g63540), *H. sapiens* (AL732446.4), *Xenopus tropicalis* (NM_001016296.1) and *Oryza sativa* (XM_472298.1) BLAP75 homologues. Identical aminoacids (aa) are boxed in black whereas similar aa are boxed in grey. The amino acids underlined in yellow represent the two conserved domains of the BLAP75 protein family. The region shown in *H. sapiens* BLAP75 (aa 151 to 211) to be necessary for binding to BLM and TopoIIIα [25] is underlined in red. The asterisk indicates the conserved lysine necessary for interaction with TopoIIIα [25].

In a screen for *A. thaliana* T-DNA (*Agrobacterium tumefaciens* transferred DNA) insertions that generate meiotic mutants, we isolated a mutant (line FCN288) disrupted in the *A. thaliana* predicted open reading frame, At5g63540 (see Materials and Methods), annotated as a protein of unknown function in TAIR (http://arabidopsis.org/). Another insertion line in At5g63540 available in the public databases (http://signal.salk.edu/), SALK_093589, was obtained (Figure 1A) and showed the same meiotic phenotype as FCN288 (Figure 1A). Genetic tests confirmed that these two mutations were allelic (see Materials & Methods), demonstrating that disruption of At5g63540 is responsible for the mutant phenotype observed in both lines. Interestingly, other insertion lines which we investigated (Salk_005449, Salk_054053, Salk_054062 and Salk_094387, Figure 1A) that contained a T-DNA insertion in the 3′ region of At5g63540 did not show any detectable phenotype (not shown). According to the T-DNA insertion sites, these mutant lines are expected to produce a truncated protein, suggesting that the C-terminal part of the protein is not necessary for its function.

The At5g63540 cDNA encodes a 644-amino acid (aa) protein (Figure 1B). Database searches using the BLASTP program (Blosum 45) for proteins similar to that encoded by At5g63540 revealed the existence of a conserved domain (from aa 101 to 194, e value 6×10^−20^) [38] annotated as a domain of unknown function (DUF1767, pfam08585) but found in the N-terminus of the nucleic acid binding domain of several protein families, represented mainly by the mammalian BLAP75 proteins and showing weak homology with an OB-fold domain [19]. When BLAST searches against the non-redundant database with the At5g63540 protein sequence were carried out, the highest scores (outside the plant kingdom) were obtained with several sequences similar to the protein BLAP75/Rmi1, including similarities outside the DUF1467 region (Figure 1B). Alignment of these proteins revealed two conserved domains: one spanning from aa 101 to aa 294 (DUF1467, Figure 1B) and another one from aa 484 to aa 627. Recent biochemical studies performed on the human BLAP75 protein showed that aa 151 to aa 211 (contained in DUF1467, and corresponding to aa 219 to aa 279 on At5g63540) are necessary for the interaction of BLAP75 with BLM and TopoIIIα. One conserved lysine (K166, corresponding to K235 in At5g63540, Figure 1B) is absolutely necessary not only for interaction with BLM-TopoIIIα but also for enhancing dHJ dissolution and HJ dissociation [25]. These authors also identified a single strand DNA binding activity domain lying in the C terminus of the human protein [25]. It corresponds to the second conserved domain of this protein family which is found in plant BLAP75 (Figure 1B).

The *S. cerevisiae* Rmi1 protein is much shorter than higher eukaryotic BLAP75, containing only the N-terminal region [22] and when it was used to query the *A. thaliana* non-redundant accessions using PSI-BLAST (Blosum 45), no hits were obtained. When the same search was carried out using *Homo sapiens* BLAP75, however, we picked up At5g63540 after the first round of iteration (e value = 8e-14, Identities = 54/187 (28%), Positives = 93/187 (49%)). We therefore called this new *A. thaliana* gene *BLAP75/RMI1*. The insertional line FCN288 (accession Ws) was named *blap75-1*, and the Salk_093589 line (Col-0 accession) *blap75-2*. This BLAST search also resulted in a high score with the *A. thaliana* At5g19950 gene (e value = 1e-11, Identities = 32/78 (41%), Positives = 47/78 (60%)). However, the GABI-Kat Line 679A11 with an insertion in At5g19950 did not display a vegetative or reproductive phenotype.

Reverse-transcriptase PCR (RT-PCR) studies showed that *BLAP75* was expressed at low levels in roots and flower buds but not in leaves (see Figure S1).

### The *A. thaliana blap75* Mutants Are Meiosis-Defective

**Figure 2:**
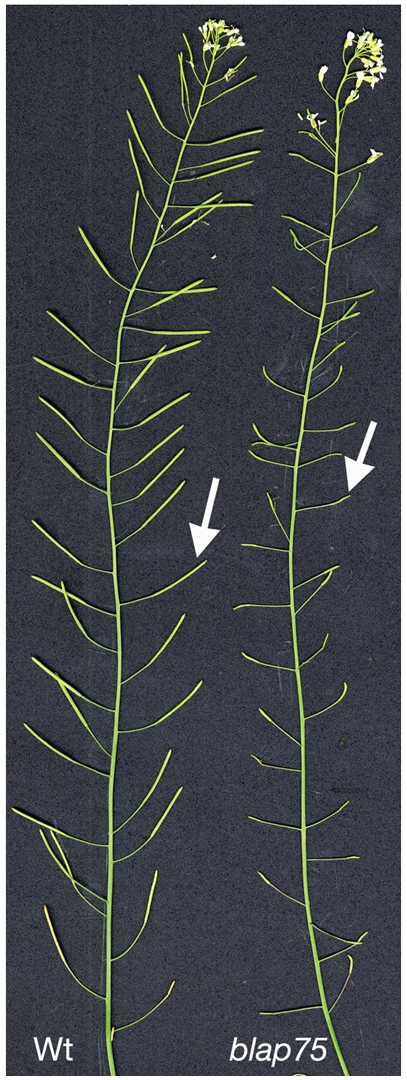
*A. thaliana blap75* mutants are sterile. Comparison of wild-type (Wt) and homozygous *blap75-1* (*blap75*) mutant plants after 30 days in the greenhouse. Arrows show siliques that elongate in wild type but not in mutant.

The two *blap75* mutants displayed the same phenotype: normal vegetative growth but short siliques (Figure 2) suggesting fertility defects. Indeed, the mean seed number per silique of both *blap75* mutants was extremely low (0.03 for *blap75-1* and 0.0006 for *blap75-2*, counted on 1,000 siliques) whereas the average is 63 and 71 seeds per silique for Ws (*blap75-1* accession) and Col-0 (*blap75-2* accession), respectively (n = 50).

**Figure 3:**
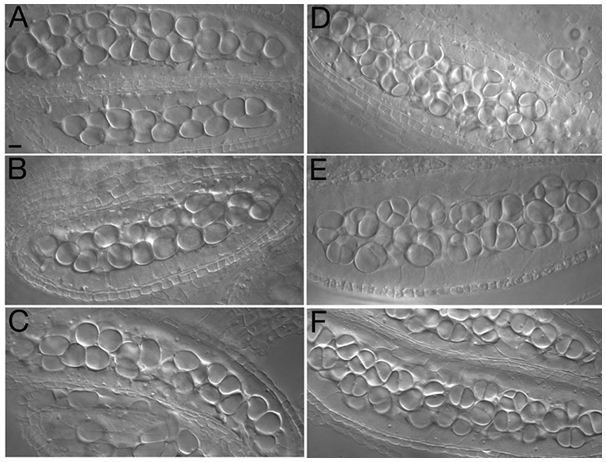
*A. thaliana blap75* mutants show defects in male sporogenesis. Male meiocytes (A–C) as well as meiotic products (D–F) are shown after anther clearing for wild type (A, D), *blap75-1* (B, E) and *blap75-2* (C, F). Bar, 10 µm.

We examined the reproductive development of these mutants and found that *blap75* sterility is due to abortion of male and female gametophytes (data not shown). No differences were seen between wild-type and mutant plants when the early stages of microsporogenesis were compared, with round pollen mother cells (PMCs) found within the anther locules (Figure 3A–C). In wild-type anthers, these cells underwent two meiotic divisions to produce a characteristic microspore tetrad (Figure 3D). Meiosis products were also detected in mutant plants but these lacked the regular tetrahedral structure, and either single, double or multiple cell products were observed (Figure 3E, F). *blap75* mutants produced a majority of dyads (55% of the cells counted for *blap75-1* (n = 348), and 73% of the cells for *blap75-2* (n = 316)) suggesting that the meiotic program is disrupted in *blap75* mutants.

**Figure 4:**
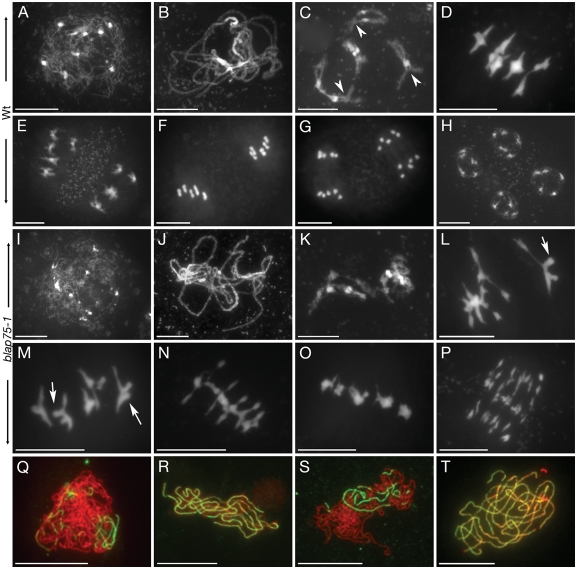
Meiotic phenotype of *blap75* mutants. (A–P) DAPI Staining of wild-type (Ws, A–H) or mutant (*blap75-1*, I–P) PMCs during meiosis. (A, I) Leptotene, (B, J) pachytene, (C, K) diakinesis, (D, L–N) metaphase I, (E) telophase I, (F) metaphase II, (G) anaphase II, (H) telophase II, (P) anaphase I. (Q–T) Co-immunolocalization of ASY1 (red) and ZYP1 (green) in wild-type (Ws, Q–R) and mutant (*blap75-1*, S–T) PMCs. Bar, 10 µm.

We therefore investigated male meiosis by staining PMC chromosome spreads with 4′,6-diamidino-2-phenylindole (DAPI). Wild-type *A. thaliana* meiosis has been described in detail in [39], and the major stages are summarised in Figure 4 (A–H). During prophase I, meiotic chromosomes condense, recombine, and undergo synapsis, resulting in the formation of five bivalents, each consisting of two homologous chromosomes attached to each other by sister chromatid cohesion and chiasmata, which become visible at diakinesis (Figure 4C, arrow heads). Synapsis (the close association of two chromosomes via the synaptonemal complex (SC)) begins at zygotene and is complete by pachytene (Figure 4B), by which point the SC has polymerised along the whole length of the bivalents. At metaphase I, the five bivalents are easily distinguishable (Figure 4D). During anaphase I, each chromosome separates from its homologue, leading to the formation of dyads corresponding to two pools of five chromosomes (Figure 4E–F). The second meiotic division then separates the sister chromatids, generating four pools of five chromosomes (Figure 4G–H), which give rise to a tetrad of four microspores (Figure 3D).

In *A. thaliana blap75* mutants, the early stages of meiosis could not be distinguished from wild type: chromosomes condensed and synapsis of the homologous chromosomes proceeded normally (shown for *blap75-1* allele in Figure 4 I–J). To confirm that no synapsis defects could be detected in *blap75* mutants we performed immunolocalization studies by double-labelling wild-type and mutant PMCs with anti-ZYP1 (a major component of the central element of the SC, [40]) and anti-ASY1 (a protein associated with the axial element of the SC, [41]) antibodies. We could not detect any difference in mutant compared to wild-type cells (Figure 4Q–T), either in the progression (Figure 4Q and 4S) or completion of synapsis (Figure 4R and 4T). However, chromosomal abnormalities appeared later at prophase, when condensing bivalents could be recognised (diakinesis, Figure 4K). At this stage in wild type, the five bivalents can be identified, each of them composed of a pair of homologous chromosomes connected one to the other where COs have occurred (some of these chiasmata are shown by arrowheads in Figure 4C). In *blap75* mutants, chromosome arms are visible but it was impossible to distinguish a chromosome arm from its homologue as if the two were intimately linked (compare 3K to 3C). At metaphase I, abnormalities were even more obvious, with a range of phenotypes illustrated in Figure 4L to 4O. In the majority of the metaphase I cells (86% n = 76 for *blap75-1* and 89% n = 107 for *blap75-2*) chromosomes did appear to be associated in bivalent-like structures because five entities can be recognised (Figure 4M–O). Nevertheless, their shape is very unusual, often showing (52% of the metaphase for each allele) bubble-like extensions (Figure 4L, M arrows) that sometimes seem to connect the bivalents together, leading to the whole set of chromosomes having a rattle-like structure (Figure 4N). In the remaining cells, the bivalent-like entities displayed a very unusual compact appearance (Figure 4O), and we never observed the five typical bivalents observed during wild-type metaphase (Figure 4D). Next, anaphase I proceeded and led to dramatic chromosome fragmentation (Figure 4P). Nevertheless, chromosome migration occurred and was followed by de-condensation of the various DNA pools produced after anaphase I. Typical telophase I could be recognised (data not shown) but meiotic division appeared to stop at this stage, and we could never identify a second meiotic division in any of the two *blap75* mutants.

When we analysed female meiosis in *blap75* mutants we observed the same defects as for male meiosis (Figure S2).

### *A. thaliana* BLAP75 Is Not Necessary for Homologous Bivalent Formation

**Figure 5:**
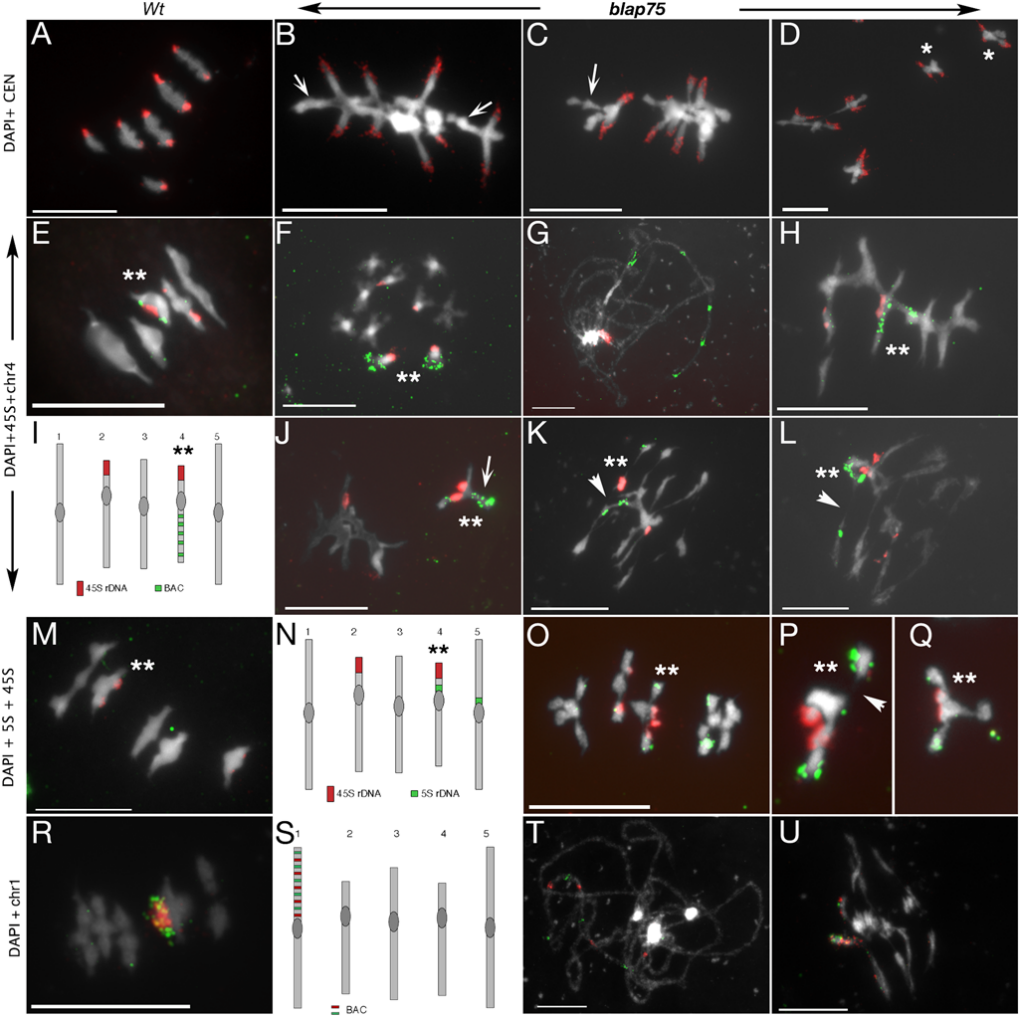
Homologous bivalents are formed in *A. thaliana blap75* mutants. (A–D) FISH on wild-type (Wt, A) or mutant (*blap75-1*, B–D) metaphase I cells with a probe directed against centromeric repeats (CEN, red). Arrows indicate the floating chromosome arms. Asterisks indicate the bivalents with more than two signals. (E–L) FISH with a 45S rDNA probe (red) and chromosome 4 long arm BACs (green) on wild-type (Wt, E) or mutant cells (*blap75-1*, F–L). (E, H, J) metaphase I, (G) pachytene stage, (K–L) anaphase I. Double asterisks indicate chromosome 4s that can be recognised with its short arm labelled in red and its long arm labelled in green, as shown on the schema in I, and in a somatic cell (F). (M–Q) FISH with probes directed against 45S (red) and 5S (green) rDNA on metaphase I cells. Double asterisks indicate chromosome 4s that can be identified by double labelling of its short arm: at the pericentromeric region in green and at the subtelomeric region in red, as shown on the schema in N. (R–U) FISH with probes directed against chromosome 1 BACs on metaphase chromsome (R), pachytene (T) or anaphase I (U) cells. All FISH preparations were DAPI-stained. Arrowheads indicate chromosome fragmentation. Bar, 10 µm.

In order to understand the nature of the metaphase structures observed in *A. thaliana blap75* mutants at metaphase I, we performed fluorescent *in situ* hybridization (FISH) analyses on PMCs with diverse probes. Firstly, we used a probe corresponding to the *A. thaliana* centromere repeat sequences (Figure 5A–D). This probe allows the very clear positioning of the ten *A. thaliana* centromeres, which were observed in wild-type and in most *blap75* cells, as expected, grouped in two pools of five, pointing toward the two spindle poles (Figure 5A–C). It also showed that contrary to what occurs in wild type, the chromosome arms are floating on the metaphase plate (Figure 5B–C, arrows), explaining the rattle-like structures seen in Figure 4N. When probed with the centromeric repeat, the “compact” *blap75* bivalents shown in Figure 4O (here in Figure 5D) appeared to have the same structure as in 5B and 5C with two centromeres directed towards opposite directions and two chromosome arms floating, except that the whole structure is more condensed (compare Figure 5D to Figure 5B,C). In some cases however, more than two centromere signals could be observed (asterisks, Figure 5D), suggesting that these entities underwent premature sister centromere uncoupling.

We also carried out FISH experiments using probe mixes designed to specifically label pairs of chromosomes: a 45S rDNA probe together with a cocktail of chromosome 4 BACs, shown in Figure 5E to L; a mixture of 45S and 5S rDNA repeats, shown in Figure 5 M to 5Q; and a mixture of chromosome 1 BACs shown in Figure 5R to U. These combinations allowed the clear identification of either chromosomes 2 and 4 (Figure 5E–L), chromosomes 2, 4 and 5 (Figure 5M–Q) or chromosomes 1 (Figure 5R–U).

Labelling of a *blap75-1* pachytene cell (Figure 5G, 5T) showed that the multiple BAC probes were correctly positioned along the chromosome arms, demonstrating that in *blap75,* synapsis is occurring between homologous chromosomes. When metaphase I PMCs were probed, we found that homologous chromosomes were associated together in bivalent-like structures (Figure 5H, J, O, P, Q) as in wild-type cells (Figure 5E, M or R). We also observed that in many cases chromosome arms are much less compact than in wild type (compare Figures 5H, J, Q to Figure 5E, M), float around, and sometimes appeared connected to each other (Figure 5H). Furthermore we observed very frequent evidences of chromosomal fragmentation (Figure 5 arrowheads).

Therefore, we can conclude from these results that the structures observed at metaphase I in *blap75* mutants are bivalent-like in the sense that they connect homologous chromosomes from pachytene to anaphase I. Nevertheless, the architecture is highly perturbed with chromosome arms floating on the metaphase plate and numerous evidence of chromosome breakages as early as metaphase I/anaphase I transition.

### *A. thaliana* BLAP75 Is Necessary for Meiotic DSB Repair using Homologous Chromosomes or Sister Chromatids as Templates

**Figure 6:**
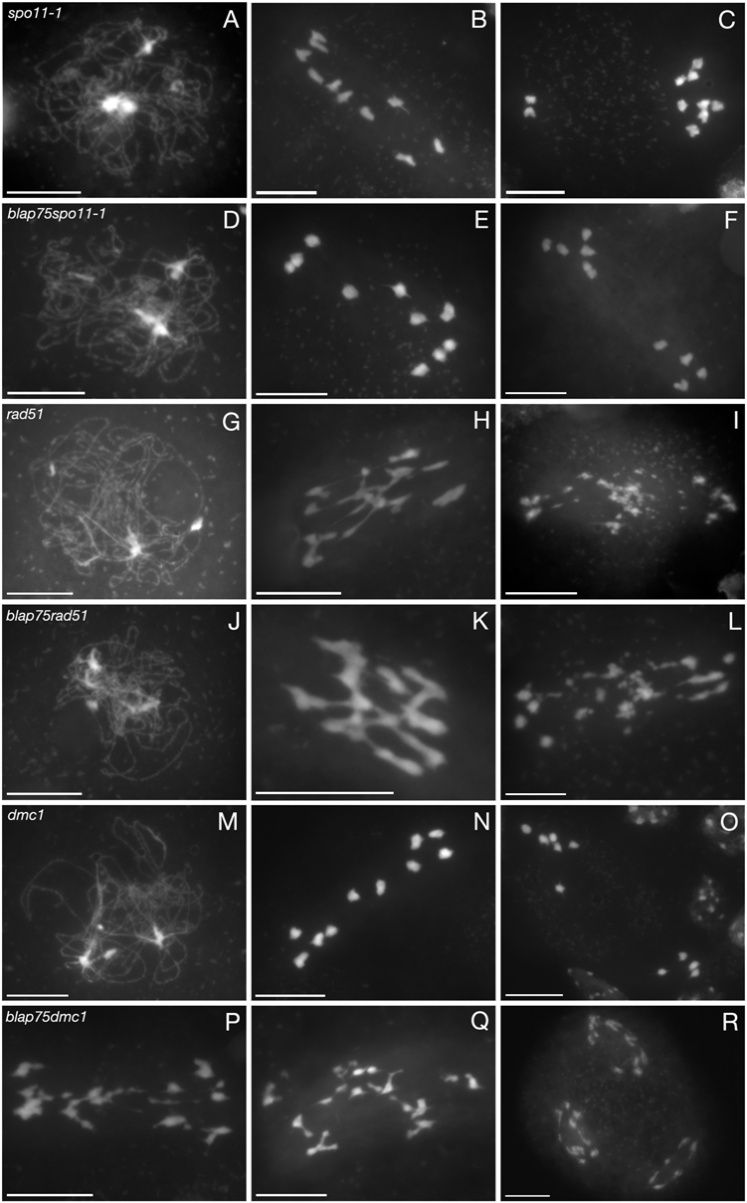
The *blap75* meiotic phenotype depends on *SPO11-1*, *RAD51* and *DMC1*. DAPI staining of male meiocytes of *spo11-1-2* (A–C), *blap75-1spo11-1-2* (D–F), *rad51* (G–I), *blap75-1rad51* (J–L), *dmc1* (M–O), *blap75-1dmc1* (P–R) at prophase I (A, D, G, J, M), or metaphase I/anaphase I transition (B, E, H, K, N, P, Q), end of anaphase I (C, F, I, L, O) or second meiotic division (R). Bar, 10 µm.

Meiotic recombination is initiated by the formation of DNA DSBs that are catalysed by Spo11 in budding yeast and in all other eukaryotes studied to date [42]. In *A. thaliana*, the disruption of *SPO11-1* or *SPO11-2* induces a typical asynaptic phenotype (Figure 6A–C) associated with a dramatic decrease in meiotic recombination, leading to the formation of achiasmatic univalents, which is correlated with an absence of meiotic DSBs [43],[44]. In order to understand if the meiotic chromosomal defects observed in *blap75* mutants were dependent upon DSB formation, we generated *spo11-1blap75* double mutants (Figure S3). These plants showed a typical *spo11-1* phenotype: synapsis failed to engage (Figure 6D), there was an absence of bivalents (Figure 6E) and lack of chromosome fragmentation at anaphase I (Figure 6F) or II (not shown). Therefore, *blap75* bivalent-like structures as well as *blap75* fragmentation are dependent upon meiotic DSB formation.

**Figure 7:**
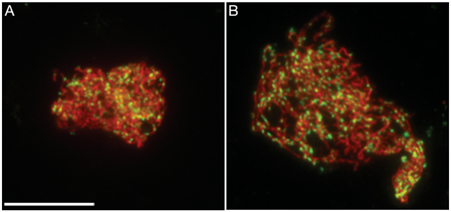
Early recombination events are not altered in *blap75*. Co-immunolocalization of ASY1 (Red) and DMC1 (green) in wild-type (Ws, A) and mutant (*blap75-1*, B) PMCs. Bar, 10 µm.

Next, we analysed the nuclear distribution of the DMC1 protein, which is an essential component of the recombination machinery (Figure S3). Its appearance on meiotic chromosomes during prophase is thought to mark the sites of recombination repair. To follow DMC1 focus formation throughout meiosis, co-immunolocalisation was performed with antibodies that recognise the meiotic protein ASY1. Detailed analysis of DMC1 progression in wild-type *Arabidopsis* meiotic prophase was described in [45]. DMC1 foci appear at late leptotene/early zygotene reaching an average of 240 foci per nucleus (239 +/− 74 n = 49) and disappear by pachytene [45]. DMC1 foci had similar characteristics in *blap75-1* male meiocytes, with an average of 235+/−68 per zygotene nuclei (n = 60) (Figure 7). Therefore, early DSB repair events do not appear to be disrupted in *blap75* mutants.

In order to obtain more precise information concerning the function and position of *A. thaliana* BLAP75 in the DSB repair steps, we also generated the *rad51blap75* and *mnd1blap75* double mutants. The Rad51 protein is a recombinase that is loaded on single-stranded DNA generated after DSB processing and mediates the search for homology and invasion of an intact homologous DNA molecule [46]. The Mnd1 protein is another key actor of the strand invasion step, stimulating the activity of Dmc1 and/or Rad51 [47]. In *A. thaliana* the two mutants, *rad51* and *mnd1*, show drastic meiotic defects that can be summarised by an absence of synapsis, the formation at metaphase I of a mass of entangled chromosomes linked together by chromosomes bridges and prominent chromosome fragmentation at anaphase I [48]–[51](shown on Figure 6G–I for *rad51*). Nevertheless, these abnormalities do not prevent meiosis II from occurring, and a second round of chromosomal segregation is observed, leading to the formation of very abnormal meiotic products (not shown). The phenotype of *rad51blap75* and *mnd1blap75* double mutants could not be distinguished from that of the *rad51* or *mnd1* single mutants (shown for *rad51blap75* in Figure 6J–L), suggesting that *A. thaliana* BLAP75 acts after RAD51 and MND1 in the DSB repair cascade.

The situation in the *A. thaliana dmc1* mutant is very different because even if meiotic DSBs are formed in this background, they are completely repaired, probably using the sister chromatid as a template [52]–[55] leading to a typical asynaptic phenotype (Figure 6M–O). Therefore, we wondered whether *A. thaliana* BLAP75 is involved in this repair pathway and we analysed the phenotype of the *blap75dmc1* double mutant. In this background we observed a cumulative effect of the two mutations. Firstly, no trace of synapsis was observed during prophase (not shown) as is the case in *dmc1* (Figure 6M). Then, at metaphase/anaphase I, chromosomes with very altered morphology, showing fragmentation and chromosome bridges, were observed (Figure 6P,Q). This fragmentation was even more spectacular while anaphase I proceeded, but second division figures were observed (Figure 6R), contrary to the situation in the *A. thaliana blap75* single mutant. Another striking difference between both genotypes was the absence in the double mutant of any bivalent-like structures at metaphase I. Therefore it appears that in the absence of BLAP75, the repair of meiotic DSBs onto sister chromatids is altered. Furthermore, bivalent-like structures formed in *blap75* mutants are dependant upon DMC1 function.

### Bivalent-Like Structures in *A. thaliana blap75* Backgrounds Are Independent of ZMM Proteins

**Figure 8:**
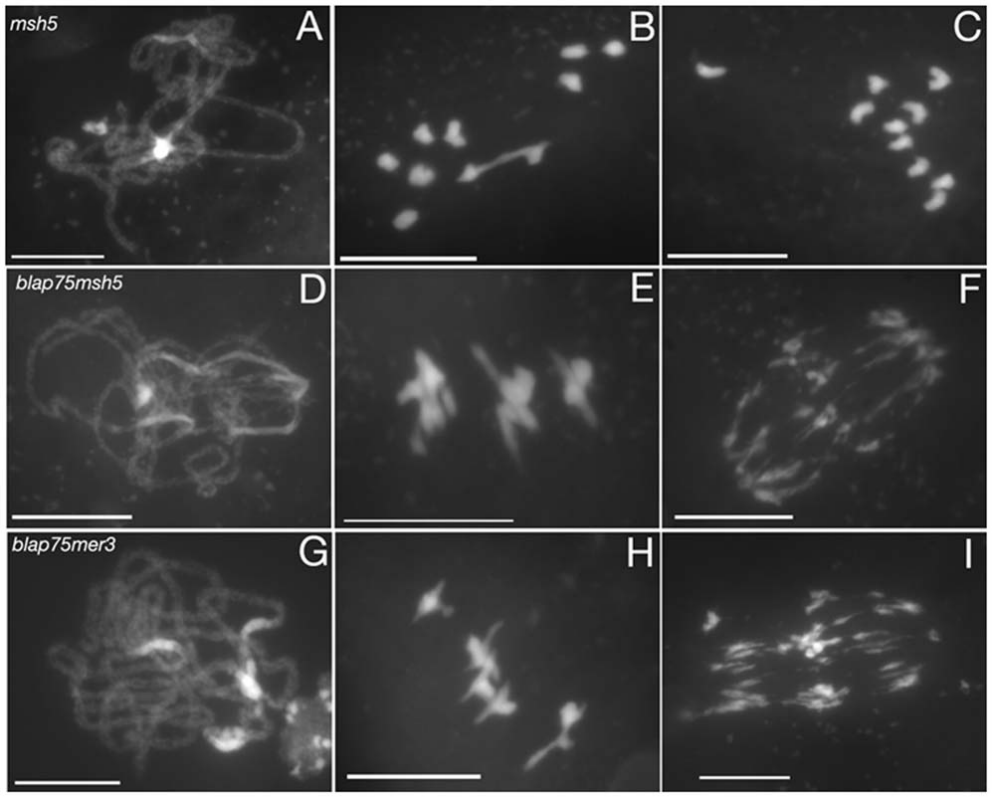
Bivalent-like structures are formed in the absence of *MSH5* or *MER3*. DAPI staining of male meiocytes of *msh5* (A–C), *blap75-1msh5* (D–F), *blap75-1mer3* (G–I) at pachytene (A, D, G), metaphase I (B, E, H) and anaphase I (C, F, I). Bar, 10 µm.

In order to understand the nature of the association between homologous chromosomes existing in *blap75* mutants, we analysed the involvement of two ZMM proteins: MSH5 and MER3 (Figure S3). Both were previously shown to be involved in the maturation of class I COs, which represent 85% of the total CO number in *A. thaliana*. Their mutation has no effect on early meiosis events but results in a highly pronounced decrease in CO formation (85% of the wild-type level for *A. thaliana msh5* and 76% for *A. thaliana mer3*) leading to a large number of achiasmatic univalents at metaphase I ([56],[57], shown for *msh5* in Figure 8A–C). When we analysed *blap75msh5* and *blap75mer3* double mutants, we could not detect any difference between them and the single *blap75* (Compare Figure 8 D–I to Figure 4J, M and P).

Therefore, we can conclude that the formation of stable associations between homologous chromosomes observed in *blap75* mutants does not require ZMM proteins.

## Discussion

### Is the BTB/RTR Complex Conserved in Plants?

In human cells and *S. cerevisiae,* BLAP75/Rmi1, BLM/Sgs1 and TopoIIIãTop3 were demonstrated to interact [19]–[22], to form one or several complexes involved in maintaining genome stability [27]. In yeast, Rmi1 and Top3 appear to act in the same pathway downstream of Sgs1 since most of the defects exhibited by *top3* and *rmi1* mutants are suppressed by mutation of *SGS1* [22],[58],[59]. These data led to the hypothesis that the yeast RecQ helicase activity (Sgs1) produces toxic DNA structures that are removed by the combined action of Top3 and Rmi1. In *A. thaliana*, the existence of this complex has not yet been shown, but the results of several recent studies provide evidence for its conservation. Firstly, the *A. thaliana recq4A-4* mutation suppresses (at least partially) the lethality of the *A. thaliana top3α-1* mutation [16]. Secondly, *A. thaliana blap75/rmi1* mutants as well as *recq4A-4* and the leaky *top3α -2* show hypersensitivity to the same DNA damaging agents as well as increased rates of somatic homologous recombination [60]. Therefore, the existence of a plant BTB/RTR complex composed of *A. thaliana* RECQ4A, TOP3α and BLAP75/RMI1 that would be involved in vegetative cell cycle surveillance, is very likely. However, its function during meiosis is less clear. Our data together with the those of [60] clearly show that two members of the plant BTB/RTR complex (BLAP75/RMI1 and TOP3α) are involved in meiotic recombination where they are likely to act in the same pathway. In *A. thaliana,* the topoisomerase 3α protein is essential for somatic development [16] making its function during meiosis difficult to investigate. Nevertheless, partial suppression of the *top3α -1* somatic phenotype by the *recq4a* mutation, together with analysis of a leaky *top3α-2* allele made it possible to show that *blap75/rmi1*, *top3α-2* and *recq4A-4top3α-1* have the same meiotic defects [60] suggesting that TOP3α and BLAP75/RMI1 act together during meiosis. Several findings, however, suggest that the last member of the complex, *RECQ4A*, may not be involved in meiosis. Firstly, its disruption does not impair meiosis [16],[17],[60]. Secondly, *recq4A-4* suppresses (at least partially) the somatic phenotype of the *top3α-1* mutation [16], but not the meiotic phenotype [60]. This suggests that if *Arabidopsis* BLAP75 and TOP3α do act together with a meiotic helicase, it is probably not RECQ4A.

### The *Arabidopsis BLAP75/RMI1* Homologue

By characterising the *A. thaliana* homologue of BLAP75/RMI1, we could report one of the first studies of the role played by a member of the BTB/RTR complex in meiosis. The BLAP75/Rmi1 proteins share a N-terminal domain containing a putative OB (oligonucleotide/oligosaccharide binding)-fold which is responsible for the single stranded DNA binding activities of proteins such as RPA or BRCA2 [61]. Nevertheless, to date, a DNA binding capacity was not associated with this region in the BLAP75/Rmi1 protein family. However it was recently shown, *in vitro,* to be necessary for *H. sapiens* BLAP75 to form a complex with BLM and hTopoIIIα and to activate the dissolution activity of this complex. The C-terminal region of BLAP75 proteins is specific to higher eukaryotes: it is not found in *S. cerevisiae* Rmi1 which is much shorter than the vertebrate or plant BLAP75. We found that mutations that disrupt this C terminal region of the *A. thaliana* BLAP75 do not lead to any detectable phenotype, at least at the reproductive level. Since in the *H. sapiens* BLAP75, the C-terminal domain was recently shown to bind to single stranded DNA *in vitro* [25], it suggests that, in *A. thaliana*, the single strand DNA binding capacity of BLAP75 is dispensable for its meiotic function. I*n vitro* studies comparing the activity of a truncated BLAP75 protein containing only the N-terminal region and the full length protein might help understand the function of this conserved higher eukaryote C-terminus extension.

### *A. thaliana* BLAP75 Is a Key Protein of the Meiotic Homologous Recombination Machinery Dispensable for Homologous Chromosome Recognition and Synapsis and Whose Disruption Allows Bivalent Formation in the Absence of ZMM Proteins

Our study of *A. thaliana blap75/rmi1* insertional mutants showed that this gene is crucial for meiosis. Disruption of *A. thaliana BLAP75* led to drastic chromosome fragmentation at anaphase I and to an absence of the second meiotic division. Inhibition of meiosis II is either indirect and due to the strong chromosomal fragmentation observed at meiosis I or *A. thaliana* BLAP75 is directly involved in meiosis II induction, but further studies are needed to decipher the precise explanation. Our study revealed that *A. thaliana* BLAP75 is involved in meiotic recombination. Firstly, we showed that the *A. thaliana blap75* meiotic phenotype depends on SPO11-1 and therefore on DSB formation. Thus it is likely that the DNA fragmentation observed in *blap75* mutants reflects DSB repair defects. Secondly, we showed that *A. thaliana* BLAP75 is not necessary for homologue recognition and synapsis. Even if we cannot exclude subtle effects (timing differences for example), major perturbations to homologous recognition, association and synapsis can be ruled out: all pachytene stages appeared perfectly normal in terms of synapsis (observed by DAPI spreads as well as ZYP1 immunolabelling) and homology (according to FISH results). Therefore, homologue recognition and synapsis occur normally in the absence of *A. thaliana* BLAP75.

Even in the absence of *A. thaliana* BLAP75, homologous bivalents are formed and can be observed from diakinesis to metaphase I. We observed that these bivalents are formed independently of two *A. thaliana* ZMM proteins (MSH5 and MER3), that are known to be necessary for all (MSH5) or most (MER3) Class I CO formation (see Introduction, Figure S3, and [56],[57],[62]). The biochemical function of ZMM is still poorly understood, but data obtained in yeast support the idea that these proteins allow the formation of stable SEI intermediates, committing these to the Class I pathway [63],[64]. Thus these proteins act just after the invasion step by the Rad51-Dmc1-coated single stranded DNA, to stabilise the newly formed heteroduplex in order to direct repair toward dHJ intermediates and CO formation. In the absence of these proteins, a stable heteroduplex cannot occur. Since homologous bivalents are formed in *blap75mer3* or *blap75msh5*, we conclude that in *blap75* mutants, recombination intermediates exist that are stable enough to form bivalent structures, even when ZMM are absent. Such intermediates could be, for example, the complex joint molecules corresponding to several interconnected DNA duplexes that have been observed in yeast *sgs1* mutants [30]. Our findings are also in agreement with data from [30],[31] who detected an anti-CO effect of Sgs1 in *zmm* mutants. It was suggested that yeast Zmm protect recombination intermediates from dissolution by Sgs1. When Zmm are removed, the anti-CO activity of Sgs1 occurs and recombination intermediates do not produce COs but are repaired onto the sister chromatid, explaining the decrease in COs observed in these backgrounds. However, when both Zmm and Sgs1 are removed, CO recombination intermediates are formed and the CO level is close to that of wild type [30],[31]. In the case of *A. thaliana*, the removal of both ZMM and BTB/RTR complex activity (*blap75* mutants), did not restore CO formation (since normal bivalents were not observed), but recombination nevertheless progressed, allowing the formation of stable bivalent-like structures.

### What Role Does BLAP75 Play in Meiotic Recombination?

Mutations affecting the BTB/RTR complexes lead to genomic instability at mitosis and meiosis. During mitosis, these mutations provoke a characteristic “hyper-rec” phenotype showing that this complex acts to suppress CO formation, likely acting at different steps of the recombination process, one of the most documented steps being the dissolution of dHJ intermediates toward NCO events [9],[23],[24],[26],[65]. This complex could also be involved in repressing recombination by acting on earlier intermediates [27], by destabilising the Rad51 filament (as was shown *in vitro* for BLM protein [10]), or by disrupting D-loop intermediates (shown also for the BLM protein, [7]). Biochemical studies have shown that BLAP75/Rmi1 has affinity for a number of DNA structures, with a preference for HJ [21]. The role of BLAP75/Rmi1 in the BTB/RTR complex would be to promote BLM-dependent dissolution of homologous recombination intermediates by recruiting TopoIIIα [9],[23],[24],[26].

Mutations affecting any member of the BTB/RTR complex also affect meiosis, but are not accompanied by a “hyper-rec” phenotype (see Introduction). Studies we performed on the *A. thaliana* BLAP75 homologue showed that abnormalities in *blap75* mutant meiosis appear at diakinesis. Homologous bivalents were formed but showed very abnormal structures, with no visible chiasmata but intimately linked homologous chromosomes arms. The recombinases Rad51 and Dmc1 act very early during meiotic recombination (Figure S3). They are loaded onto 3′ single strand DNA generated at DSB sites and are thought to play a crucial role in the search for homologous intact DNA duplexes. Mnd1, together with its partner Hop2, was shown to stabilise the Rad51 presynaptic filament and to promote D-loop formation [66]. We analysed the phenotypes of the *rad51blap75* and *mnd1blap75* double mutants and found that we could not distinguished them from single *rad51* or *mnd1*, showing that the *blap75* phenotype depends not only on SPO11-1 (as discussed earlier), but also RAD51 and MND1. We also found that DMC1 focus number was identical in *blap75* and in wild type. The Dmc1 protein is a meiotic specific RecA homologue that plays a crucial role in driving meiotic DNA repair towards the homologous chromosome instead of the sister chromatid. If BLAP75 was involved in destabilizing early recombination intermediates, one might expect to see a difference in DMC1 focus formation. Since this difference was not observed, it suggests that BLAP75 is not involved in destabilising early recombination intermediates. Therefore, *A. thaliana* BLAP75/RMI1 is a protein necessary for the repair of meiotic DSBs that acts after the invasion step mediated by RAD51 and associated proteins. We also showed that BLAP75 is necessary for repair onto sister chromatids, since DSB repair in the *dmc1* background is perturbed in the absence of *BLAP75*. Therefore, taken together all these data suggest that BLAP75, probably along with TOP3α, fulfils a key function in meiotic recombination by processing (dissolving) recombination intermediates, which are dependent upon RAD51, MND1 and DMC1 and generated during meiotic DSB repair on either homologous chromosomes or sister chromatids.

## Materials and Methods

### Plant Material

The *blap75-1* mutant (FCN288 line) was obtained from the Versailles *Arabidopsis* T-DNA transformant collection [67]. Mutant screening was performed as described in [68]. The *blap75-2* mutant, line Salk_093689, was obtained from the collection of T-DNA mutants at the Salk Institute Genomic Analysis Laboratory (SIGnAL, http://signal.salk.edu/cgi-bin/tdnaexpress) [69] and provided by the Nottingham *Arabidopsis* Stock Centre (NASC) (http://nasc.nott.ac.uk) as well as lines Salk_005449, Salk_054062, Salk_054053 and Salk_094387.

The *spo11-1* allele used is *spo11-1-2* described in [44],[54]. The *dmc1*, *rad51-1*, *msh5*, and *mer3* mutants were described in [48],[52],[56],[57],[70].

### Genetic Analyses

Isolation of *blap75-1*: the FCN288 line segregated 3∶1 for the meiotic mutation (revealing the presence of a single recessive mutation) and 3∶1 for kanamycin resistance (one of the T-DNA markers). After crossing to wild type, linkage between the T-DNA insert and the meiotic phenotype was confirmed as described in [44].

We tested for allelism between the *blap75-1* and *blap75-2* mutations by crossing two heterozygous plants *blap75-1^+/−^* and *blap75-2^+/−^*. Among the F1 plants, one fourth was sterile and carried each of the mutant alleles.

Double mutants were obtained by crossing plants heterozygous for each mutation. The resulting hybrids were self-pollinated. We used PCR screening to select the sterile plants in the F2 progeny homozygous for both mutations.

### cDNA Studies

The full length cDNA sequence for At5g63540 was obtained from NCBI (http://www.ncbi.nlm.nih.gov/entrez/) with the accession number AY954880 and checked by RT-PCR amplification.

### Isolation of Plant T-DNA Flanking Sequences

The left border genomic sequence flanking the *blap75-1* T-DNA insert was amplified by thermal asymmetric interlaced PCR (TAIL PCR) according to [71], with the modifications described in [44]. Subsequent sequencing revealed that the insertion was at nt 696 in the AY954880 sequence. The right border could not be amplified because of a complex insertion of two T-DNAs in tandem. The T-DNA insertion led to a deletion of the 5′ region of At5g63540 (since no amplification product could be detected with primers P6 (GGAGCCCGTCTAGAAGTCGACAACGA) and P10 (GCTCACTGACTCCGACGGAT) or P3 (ACGAAGAAGAAGAAGATGAAACTGG) and P1R (TGAGTGGGCAGCCAATGTTAAC), however we checked that the gene located 5′ to At5g63540 (At5g63550) was not affected.

The left border of *blap75-2* was amplified with primers LbSalk2 and P3 and subsequently sequenced, showing that the T-DNA was inserted in At5g63540 (nt 272 of sequence AY954880). The right border could not be amplified, and we observed that the T-DNA insertion induced a deletion of AY954880 3′ region (at least from nt 272 to nt 900 of AY954880).

### Oligonucleotides for PCR Genotyping

The *spo11-1-2* mutation was identified using a CAPS marker. PCR amplification was performed with primers MG52 (GGATCGGGCCTAAAAGCCAACG) and MG96 (CTTTGAATGCTGATGGATGCATGTAGTAG) and subsequently cleaved with *Ase*I. The digestion generates two 500 bp fragments for the mutant allele only. The *blap75-1* T-DNA left border was amplified with primers P2 (GCAGCTAGAGTTGCTCTGGTTG) and LbBAR2 (CGTGTGCCAGGTGCCCACGGAATAG). The wild-type *blap75-1* allele was amplified with primers P2 and P7 (GCTGGTCCGTTTGTTCTGCAG).

*blap75-2* T-DNA left border was amplified by PCR with primers P6 and primer LbSalk2 (GCTTTCTTCCCTTCCTTTCTC). The wild-type allele of *blap75-2* was PCR amplified with primers P6 and P1R.

### Sequence Analyses

Protein sequence similarity searches were performed at the National Centre for Biotechnology Information (http://www.ncbi.nlm.nih.gov/BLAST) and the *Arabidopsis* Information Resource (TAIR, http://www.arabidopsis.org/Blast), using BLOSUM45 matrix and default parameters. Sequence analyses were performed with BioEdit software (http://www.mbio.ncsu.edu/BioEdit/bioedit.html).

### Antibodies

The anti-ASY1 polyclonal antibody has been described in [41]. It was used at a dilution of 1∶500. The anti-ZYP1 polyclonal antibody was described by [72]. It was used at a dilution of 1∶500. The anti-DMC1 antibody was described in [45] and the purified serum was used at 1∶20.

### Microscopy

Comparison of early stages of microsporogenesis and the development of PMCs was carried out as described in [44]. Preparation of prophase stage spreads for immunocytology was performed according to [41] with the modifications described in [73]. The fluorescence *in situ* hybridization (FISH) was performed according to [74]. The *A. thaliana* 180 bp pericentromeric tandem repeat (pAL1, [75]) and pTa71, a 9 kb clone containing 18S-5, 8S-25S *Triticum aestivum* rDNA [76] were labelled by biotin nick translation mix according the manufacturer's instruction (Roche) and detected by Avidin-Texas Red and goat anti-avidin-biotin antibodies (Vector laboratories). A 3.5 kb fragment of 5S *A. thaliana* rDNA (pCT4.2, [77]) and seven BACs (F25I24, F25E4, F28A21, F17L22, F10M23, F4I10, F22I13) from the long arm of chromosome 4 (http://www.arabidopsis.org/servlets/mapper) were labelled by digoxigenin nick translation mix according the manufacturer's instruction (Roche) and were detected by mouse anti-digoxigenin antibodies (Roche), rabbit anti-mouse FITC and goat anti-rabbit Alexa-488 antibodies (Molecular Probes). To label the chromosome 1 arm, ten BACs (F6F3, F24B9, F14N23, F13K23, F19K19, F14010, F26F24, F17L21, F12K21, F26G16) were labelled alternately with digoxigenin-dUTP and biotin-dUTP and detected as described above.

All observations were made using a Leica DMRXA2 microscope; photographs were taken using a CoolSNAP HQ camera driven by Open-LAB 4.0.4 software; all images were further processed with OpenLAB4.0.4 or AdobePhotoshop 7.0.

## Supporting Information

Figure S1 *A. thaliana BLAP75* mRNA expression in different plant tissues.
(0.13 MB DOC)

Figure S2 *A. thaliana blap75* mutants show defects in female meiosis.
(0.40 MB DOC)

Figure S3 Schematic representation of the different steps of meiotic recombination investigated in this study.
(0.06 MB DOC)
