# Supplementary material for: The Arabidopsis BLAP75/Rmi1 Homologue Plays Crucial Roles in Meiotic Double-Strand Break Repair
Source: PLoS Genet. 2008 Dec 19;4(12):e1000309. doi: 10.1371/journal.pgen.1000309 (PMC2588655; doi:10.1371/journal.pgen.1000309)
Supplement: Figure S1 — A. thaliana BLAP75 mRNA expression in different plant tissues. (0.13 MB DOC) [file pgen.1000309.s001.doc]

Figure S1

A

B

Figure S1 legend and method:

RT-PCR on mRNA isolated from leaves (Le), roots (R) and flower buds (B) of wild-type plants. cDNA synthesis was performed with Superscript RT (Invitrogen, http://www.invitrogen.com) on total RNA (3 μg).

A: cDNA were calibrated according to the expression of the adenine phosphoribosyltransferase-encoding gene (*APT*, Moffat et al. 1994, Gene. 143: 211-216), after 30 amplification cycles at 60°C.

B: *BLAP75* amplification was obtained after two rounds of nested PCR, first with primers P11 and P18 (25 cycles at Tm= 60°C), and second with primers P9 and P15 (30 cycles at Tm= 60°C).

L : Fermentas 1 kb DNA ladder

Δ1: PCR I water control

Δ2: PCR II water control

Primer sequences:

APT1: TCCCAGAATCGCTAAGATTGCC

APT21: CTCAATTACGCAAGCAC

P9: CCCACTCAAAGGGAGGTATGA
P11: GAAGAAATGCGTAGACGGCGC

P15 : GAGGTTCATCCATCGCCCGT

P18: TGAGACAACTCGGCTAAGT
