# Supplementary material for: The Arabidopsis BLAP75/Rmi1 Homologue Plays Crucial Roles in Meiotic Double-Strand Break Repair
Source: PLoS Genet. 2008 Dec 19;4(12):e1000309. doi: 10.1371/journal.pgen.1000309 (PMC2588655; doi:10.1371/journal.pgen.1000309)
Supplement: Figure S2 — A. thaliana blap75 mutants show defects in female meiosis. (0.40 MB DOC) [file pgen.1000309.s002.doc]

Figure S2 :


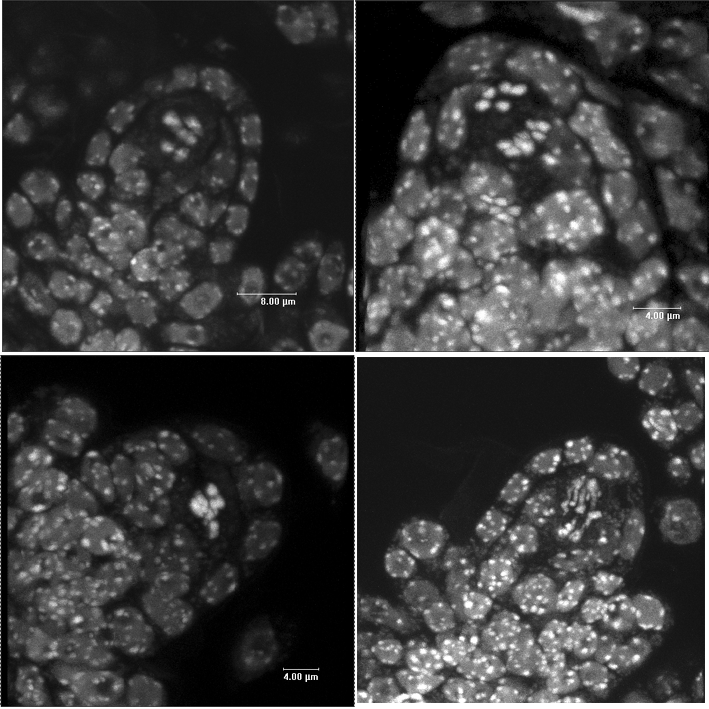


**A**

**B**

**C**

**D**

Comparison of meiosis in developing ovules of wild-type (A-B) or *blap75-1* mutant (C-D) plants after propidium iodide staining.

The five *Arabidopsis* bivalents can be identified at metaphase I in wild type (A) but not in *blap75* mutant (C). At anaphase I, chromosomal segregation leads to dyad in wild type (B) whereas drastic chromosomal fragmentation is observed in *blap75* mutant (D).

Sample preparation for confocal laser microscopy was performed as described in Motamayor et al. (2000) Sex. Plant Reprod. 12: 209-218.
