# Supplementary material for: The Arabidopsis BLAP75/Rmi1 Homologue Plays Crucial Roles in Meiotic Double-Strand Break Repair
Source: PLoS Genet. 2008 Dec 19;4(12):e1000309. doi: 10.1371/journal.pgen.1000309 (PMC2588655; doi:10.1371/journal.pgen.1000309)
Supplement: Figure S3 — Schematic representation of the different steps of meiotic recombination investigated in this study. (0.06 MB DOC) [file pgen.1000309.s003.doc]

Figure S3

Figure S3 legend:

Schematic representation of the different steps of meiotic recombination.

Mutants used in this study are indicated as well as their effect on meiotic recombination and their meiotic defects.
